# Configurations of actual and perceived motor competence among elementary school children in China: differences in physical activity

**DOI:** 10.3389/fpubh.2023.1280643

**Published:** 2023-12-19

**Authors:** Hongqin Chai, Rui Xue, Lei Yao, Miao Miao, Bochen Han

**Affiliations:** ^1^School of Education, Beijing Sport University, Beijing, China; ^2^Shanxi Youth Vocational College, Taiyuan, Shanxi, China; ^3^China Football College, Beijing Sport University, Beijing, China

**Keywords:** physical activity, school-age children, motor competence, actual motor competence, perceived motor competence

## Abstract

**Background:**

Actual motor competence (AMC) and perceived motor competence (PMC), as determinants of children’s active participation in physical activity (PA), were important for children’s healthy development. The correlation between the two had been confirmed. To further understand this relationship, this study investigated the current status of AMC, PMC, and PA in Chinese school-age children, used a person-centered approach to reveal the characteristics of the development of motor competence (MC) in Chinese school-age children and the differences in the level of PA among different MC profiles of children.

**Materials and methods:**

A total of 532 children (age: *M* = 9.37, SD = 1.80 years-old) from grades 1 to 6 participated in this cross-sectional study (male, *n* = 284, 53.4%; female, *n* = 248, 46.6%). The Test of Gross Motor Development-3 (TGMD-3) was used to measure children’s AMC, the Pictorial Scale of Perceived Movement Skill Competence (PMSC) to measure children’s PMC, and the revised Chinese version of Physical Activity Questionnaire for Older Children (PAQ-C) to assess children’s PA levels.

**Results:**

There were some gender differences in AMC but no significant gender differences in PMC and PA in children. AMC and PA levels increased as the children aged, while PMC showed some decline. Cluster analysis identified four groups of children with different MC profiles. Two groups of children had corresponding AMC and PMC levels (the “high-high” cluster, *N* = 200, 38.91%; the “low-low” cluster, *N* = 63, 12.26%), and the other two groups were inconsistent in AMC and PMC (the “high-low” cluster, *N* = 100, 19.46%; the “low-high” cluster, *N* = 151, 29.38%). Significant differences in PA levels were found between children with different MC profiles. The “high-high” cluster children had the highest PA levels, whereas the “low-low” cluster children demonstrated the lowest PA levels.

**Conclusion:**

AMC, PMC, and PA in Chinese school-age children were consistent with the pattern of child growth and development. Children with high AMC and high PMC usually had high levels of PA. Therefore, it was recommended to seize the best opportunity to intervene with children, and family, school, and community should synergize to help children improve AMC and PMC, and then actively participate in PA.

## 1 Introduction

Motor competence (MC) referred to the ability to skillfully perform physical skills and movement patterns (1). When an objective measure method was used to assess MC, it could be referred to as actual motor competence (AMC); when an individual’s assessment and perception of his or her own level of MC was used, it could be referred to as perceived motor competence (PMC) (2). In developmental psychology, AMC and PMC had an important status. Since childhood was a period of rapid development of MC, in this study, the AMC in childhood was reflected by the performance of fundamental motor skills (3), and PMC was an individual’s beliefs about performing a certain motor skill or self-perception of AMC (4). Research had shown that children’s AMC and PMC were important factors influencing children’s participation in physical activity (PA) (5–7).

An increasing number of studies had explored the relationship between AMC and PMC (8–10). Quantitative research had become the main research paradigm, and there were different voices on the research paths, one path was variable-centered and the other was person-centered. Most of the current studies had mainly used a variable-centered approach (e.g., regression analyses) to examine the relationship between AMC and PMC in children and adolescents (11–13), which described the correlation between the two variables. Most studies had found a positive correlation between AMC and PMC in children and adolescents of different ages (14, 15). A meta-analysis study also found a low to moderate strength correlation between FMS and PMC (*r* = 0.19–0.46) (10). However, some studies had also found no significant correlation between the two (16). Diao et al. (16) examined the relationship between children’s FMS and their self-perception at different ages, using the TGMD-3 to measure children’s FMS and the PMSC to measure PMC, and found that there was no correlation between FMS and self-perception in preschool children, but a lower correlation existed between FMS and self-perception in school-age children. Inconsistencies in the strength of these relationships could be due to cultural differences, variability in the level of cognitive development of children at different ages, and the use of different measurement instruments (17).

A small number of studies had used a person-centered approach. A person-centered approach allowed for the classification of subjects according to different characteristics and explored group heterogeneity that could not be distinguished by a variable-centered approach (18). Weiss and Amorose (19) first used a person-centered approach to determine whether children overestimated, underestimated, or accurately estimated their MC, identifying five clusters, but they used a teacher-reported measure of children’s AMC. Recent studies had assessed children’s AMC using objective measures, De Meester et al. (20) identified three clusters of children with different MC profiles among 6-12-year-old in the U.S. Two clusters of children had AMC and PMC levels that corresponded to each other (i.e., low-low, 34.26%, and high-high, 33.70%), and one group of children had AMC and PMC levels that were different (i.e., low-high, 32.03%). In contrast, De Meester et al. (21), in another study of Belgian adolescents aged 13–15 years, identified four groups of adolescents with different estimations of their MC profiles, with the presence of two overestimated (51%) and two accurately estimated (49%) groups, and with the overestimated MC group of adolescents having stronger motivation to participate in sports and levels in PA, especially in the case of adolescents with low AMC and high PMC adolescents. Bardid et al. (22) explored how children with different MC profiles differed in terms of motivation to exercise and overall self-worth, identifying four groups of children with different profiles, two with corresponding levels of AMC and PMC (i.e., low-low and high-high), and two with varying levels of AMC and PMC (i.e., high-low, low-high), and they found that lower PMC children demonstrated lower levels of exercise motivation and lower levels of overall self-worth, even though they had higher AMC.

PA was critical to physical health, cognitive development, and psychological and social adaptation in children and adolescents (23). Previous studies had shown that MC was one of the factors that promoted children and adolescents’ participation in games, sports, or other types of PA (24, 25). On the relationship between MC and PA, Stodden et al. (26) proposed a model of dynamic mechanisms affecting PA changes in children based on previous studies, demonstrating the relationship between AMC, PMC, PA, and obesity risk. The model assumed that AMC and PA worked synergistically to influence the weight status of children and adolescents. In a positive spiral of engagement, higher levels of AMC and PA were associated with healthy weight status and lower obesity risk, whereas in a negative spiral of disengagement, lower levels of AMC and PA were associated with unhealthy weight status and higher risk of obesity. Many studies had demonstrated a positive association between AMC and PA levels in children (27–29), and this relationship strengthens with age (30). Stodden et al. (26) also suggested that the interrelationships and dynamics of AMC and PA were mediated by factors such as healthy fitness and PMC throughout childhood (26), identifying PMC as a key intervening factor in explaining how AMC could affect PA in children (31). Related studies had shown that AMC interacted with PMC as one of the most powerful potential mechanisms influencing the motivation and persistence of children’s PA participation (32, 33). Some studies had identified AMC rather than PMC as the main factor influencing physical activity participation in children and adolescents (34, 35), but some studies had suggested that children with high AMC and PMC were more physically active and had emphasized that the development of both AMC and PMC during childhood was an important factor in increasing physical activity levels and participation (36). Therefore, AMC and PMC were potentially important factors influencing physical activity levels and participation in children and adolescents, and more research evidence were needed to confirm the role of AMC and PMC in promoting physical activity, which had positive implications for the healthy development of children and adolescents.

Although the relationship between AMC and PMC had been explored in the past literature, there were still some issues that required further research. First, most studies used a variable-centered approach. Although the variable-centered approach could yield total variable scores and the relationship between different dimensions and different outcome variables, there were still some limitations in this approach (37). The variable-centered approach made it difficult to find out which best combination of AMC and PMC contributed most to an individual’s health behavior. For children with differences in AMC and PMC (e.g., high AMC and low PMC, or low AMC and high PMC), a variable-centered approach could provide only limited revelations (33). Second, although some researchers had begun to turn their attention to person-centered approaches in recent years (20–22), they were still only a minority, especially on the developmental characteristics of children’s MC in the Chinese cultural context. The person-centered approach no longer focused on the examination of specific variables and could better reflect the comprehensive characteristics of individuals (38). This approach could reveal the relationship between the combination of different levels of AMC and PMC and PA, suggesting that different individuals had different MC profiles, which in turn allows for targeted interventions for these individuals. Finally, the results of the clustering of individual MC profiles still showed some differences (19–21). This could be related to the fact that the researchers chose different measurement tools, the cultural context in which the samples were located, and the age group of the samples. In addition, previous studies had mostly explored the relationship between MC and PA in children and adolescents from the perspective of variables, and had validated or supplemented Stodden’s model (12, 31, 39), while less exploring the performance of children’s PA levels under different MC characteristics.

Based on the above, the present study was rooted in the Chinese cultural context and adopted a person-centered approach to reveal the characteristics of MC development and differences in PA levels among Chinese schoolchildren in order to provide targeted guidance for future interventions. Specifically, this study included the following three main purposes. First, this study aimed to investigate the levels of AMC, PMC, and PA in schoolchildren aged 6–12 years old in the Chinese cultural context, and to explore the differences that exist in these variables across gender and age groups. Second, a person-centered approach was used to identify groups of children with corresponding levels or different levels of AMC and PMC. Based on developmental models (26) and previous researches (19–22), we hypothesized that four groups of children with different combinations of MC characteristics would be identified (i.e., low-low, high-high, low-high, and high-low). Third, the present study also aimed to explore how groups of children based on various MC profiles behaved differently in PA. Based on previous research (20, 35), it was hypothesized that the low-low group of children would perform the lowest level of PA, while the high-high group of children would perform the highest level of PA.

## 2 Methods

### 2.1 Participants

This study adopted stratified cluster sampling method to select a primary school from urban, rural–urban fringe and suburban areas of Beijing to ensure that the sample is balanced in terms of rural and urban distribution, and randomly select one class from grades 1–6 of each school for testing and filling in questionnaires. Before testing pupils in each elementary school, the principal of each school was contacted and permission was obtained from the school district and institutional review board in which the three elementary schools were located to conduct the study. Then, each student and their parents were given informed consent for the study. A member of the research team read the informed consent form to the students in the class, and the students answered whether they would like to participate. All the students were willing to participate in the study. After school, students would take the informed consent form of parents back home, and parents would fill in whether their children were willing to participate in the study. Informed consent from parents of all students was obtained for this study. A total of 562 children from grades 1 to 6 participated in the study, of which 20 children withdrew from the test due to physical reasons, and 10 children did not attend school to fill in the questionnaire. This study finally collected complete data of 532 students, of whom 284 were male, accounting for 53.4%; 248 girls, accounting for 46.6%; The mean age was 9.37 years (SD = 1.800 years, range from 6 to 12 years); Students had an average BMI of 18.59 (SD = 4.10); Boys’ average BMI was 19.21(SD = 4.37) and girls’ average BMI was 17.88 (SD = 3.66). The specific information of the subjects was shown in Table 1.

**Table 1:** Demographics of the participants (*N* = 532).

Characteristic	Total	Percentage
**Gender**		
Boy	284	53.4
Girl	248	46.6
**School district**		
Urban area	184	34.6
Rural–urban fringe	184	34.6
Rural area	164	30.8
**Grade**		
One	89	16.7
Two	86	16.2
Three	88	16.5
Four	90	16.9
Five	91	17.1
Six	88	16.5
Eye sight		

### 2.2 Measures

The Test of Gross Motor Development-3 (TGMD-3) was used to assess school-age children’s AMC, which was specifically designed for structured assessment of the level of development of fundamental movement skills in children aged 3–10 years (40). It had been proved that TGMD-3 had good applicability to Chinese children aged 3–12 years, and could be used as an effective tool to assess AMC of Chinese children (41). The administration of TGMD-3 involved the completion of two parts: (1) locomotor skills (run, gallop, hop, skip, slide, and horizontal jump), and (2) object control skills (two handed strike, one handed strike, catch, kick, overhand throw, underhand throw, and stationary dribble) (42). All six testers were trained prior to the test in order to master the standardized motor skills required for the demonstration and to clarify the scoring criteria, and the test is scored by on-site observation and video recording methods. Each skill assessment consisted of 3–5 standardized movement observation indicators. All test items were completed in a designated standardized field and procedure. The tester guided the subject child through each skill test 2 times. When a criterion was performed, “1” was recorded, and the opposite was recorded as “0.” The higher the score for each movement, the better the mastery of that movement. The scores of the 2 tests were aggregated into a total score assessment and the study was statistically analyzed using raw scores. The total score for locomotor skills was 46, and the total score for object control skills was 54, and the total AMC was 100 points. The tests lasted for 1 month and were all completed during physical education lessons in each class. In this study, the Cronbach’s α coefficient of the scale was 0.859, demonstrating a good reliability, and the goodness of fit (χ^2^/df = 1.157, GFI = 0.964, AGFI = 0.941, RMSEA = 0.024) showed a good validity. The formal test also included an inter-rater reliability test and a re-test reliability test. During the test, all testers scored 10 subjects at the same time, and 20% of the total number were retested 2 weeks after the first test. With a total of six testers in this study, the Kendall’s Harmony Coefficient was used to assess inter-rater reliability (43). The results showed that the Kendall’s Harmony Coefficient value for TGMD-3 test was 0.873, which was greater than 0.8 and reached the level of significance, indicating a high degree of consistency in the inter-rater scores of the test (44). The intraclass correlation efficient (ICC) was used to assess the retest reliability, and the results showed that ICC of TGMD-3 was greater than 0.75, which indicated that TGMD-3 had a good retest reliability (41).

The Pictorial Scale of Perceived Movement Skill Competence (PMSC) was adopted to assess school-age children’s PMC (45), which was confirmed to have good retest reliability, internal consistency, and construct validity in Chinese children aged 4–9 years (46). The test instrument had 2 manuals for boys and girls, and each had 13 items (6 items for perceived locomotor skills and 7 items for perceived object control skills). Each movement was presented using two cartoon pictures, one for the movement that was done well and one for the movement that was not done so well. Below each picture there were four circles, and each circle had a corresponding score, with a maximum of 4 points (e.g., 4 = I’m really good at running) and a minimum of 1 point (e.g., I’m not too good at running), depending on how well the movement was performed. The test procedure was as follows: first, the tester introduced the subject to what movement the boy/girl was doing in the picture. Second, the subject was told which boy/girl was doing the movement well and which was not so well, and the child was asked to choose the picture of the child that most resembled him/herself. Finally, the tester told the child what each circle represented and recorded the corresponding score. The mean scores of the scale were analyzed in this study. The Cronbach’s *α* coefficient of the scale was 0.844, showing a good reliability. Meanwhile, the scale had a good validity (χ^2^/df = 2.524, GFI = 0.956, AGFI = 0.932, RMSEA = 0.055) in the present study.

Children’s PA were measured using the revised Chinese version of Physical Activity Questionnaire for Older Children (PAQ-C) (47, 48). The PAQ-C employed memory trails to facilitate childrens’ recall of participation in PA, and numerous studies had shown that the questionnaire had good psychometric properties, had been shown to have good reliability, validity, and cross-cultural stability, and was applicable to the measurement of PA levels in school-age children (49, 50).

The PAQ-C questions were clear and easy to understand, and children did not need to recall detailed information about the duration and intensity of exercise, which could significantly reduce recall bias and make it suitable for large samples of people. The PAQ-C consisted of 7 question items that asked subjects to recall the number of days, time, and frequency of participation in high, moderate, and low intensity PA over the past 7 days. The scale was based on a 5-point scale, and the PA score was the average of the 7 items, with higher scores representing higher levels of PA. PAQ-C 1 surveyed children’s intensity of activity in sports such as running, basketball, and badminton (1 = 0 times, 5 = 7 or more times); PAQ-C 2–5 surveyed children’s PA level during physical education classes, after school, and on weekends (1 = low level, 5 = highest level); PAQ-C 6 surveyed children’s overall PA level in the past week in out of class time (1 = low level, 5 = highest level); PAQ-C 7 surveyed children’s everyday PA in the past week (1 = never, 5 = very often). In this study, the scale had a good reliability (Cronbach’s *α* coefficient = 0.835) and good validity (χ^2^/df = 1.8888, GFI = 0.985, AGFI = 0.971, RMSEA = 0.042).

### 2.3 Analysis

In this study, the Statistical Package for the Social Sciences (IBM SPSS Statistics for Windows, version 26.0. Armonk, NY, United States: IBM Corp.) was used for descriptive and inferential statistical analysis (with *p* < 0.05). The common method bias was tested by Harman single factor test. Taking the eigenvalue greater than 1 as the standard, principal component analysis was used to extract the common factor, and the partial correlation was obtained by isolating the first common factor (51). The results showed that the variance explanation rate of the first factor without rotation was 18.297%, which was less than the critical standard of 40%. Therefore, there was no serious common method bias in this study.

**Aim 1:** Children’s ages were divided into three stages based on their developmental stages of perceptual abilities. First, descriptive statistics were performed on the scores of AMC, PMC, and PA on the three age groups. Second, independent samples *t*-tests were performed for gender differences in the variables at each age. Finally, one-way ANOVA was performed for differences in variables across age groups, and *post hoc* comparisons were conducted using the LSD method.

**Aim 2:** Before performing the cluster analysis, the AMC and PMC scores were standardized first and univariate and multivariate outliers were removed. Eight univariate outliers (values that deviated from the mean by more than three times the standard deviation) and 10 multivariate outliers (determined using the Mahalanobis distance measure) were found. A final sample of 514 was included. Cluster analysis was performed using both hierarchical clustering and K-means clustering. First, through hierarchical clustering, the number of variables clustered was determined by looking at the spectrogram using the clustering method of intergroup linkage and squared Euclidean distance measurement intervals. The spectrograms and clustering coefficients indicated that clustering the children’s MC characteristics into four classes was more desirable and reasonable (each cluster explains at least 50% of the variance on both AMC and PMC). Second, further results were obtained by K-means clustering, setting the K value to 4, for each clustered element. Finally, the results of clustering on children’s AMC and PMC were further tested accordingly.

**Aim 3:** One-way ANOVA was used to test for differences in PA levels among groups of children with different MC profiles, and the LSD method was used for *post hoc* comparisons.

## 3 Result

As shown in Table 2, there was no significant gender difference in locomotor skills of 6–7 year old children, while object control skills (*p* < 0.01) and AMC (*p* < 0.05) of 6–7 year old boys were significantly better than those of girls; locomotor skills of 8–9 year old girls were significantly better than those of boys (*p* < 0.001), while object control skills of 8–9 year old boys were significantly better than those of girls (*p* < 0.001), and AMC of 8 to 9-year-olds AMC did not have significant gender differences; 10–12-year-old boys had significantly better object control skills than girls (*p* < 0.001), and 10–12-year-olds mobility skills and AMC did not have significant gender differences. PMC as well as the two dimensions and PA did not show significant gender differences at all ages (*p* > 0.05). From the overall view of AMC test results, the total AMC test scores of children showed a trend of gradual increase with age. Children’s PMC differed significantly across age groups, with PMC in children aged 6–7 years significantly higher than those in children aged 10–12 years. Overall, children’s PA levels gradually increased with age, with children aged 10 to 12 years having significantly higher PA levels than children aged 6–7 years.

**Table 2:** Descriptive statistics.

Variables	Full score	6 ~ 7 (A)				8 ~ 9 (B)				10 ~ 12 (C)				*F*	*p*	Multiple comparisons (LSD)
		Total (*n* = 104) *M* (SD)	Boy (*n* = 57) *M* (SD)	Girl (*n* = 47) *M* (SD)	*p*	Total (*n* = 167) *M* (SD)	Boy (*n* = 88) *M* (SD)	Girl (*n* = 79) *M* (SD)	*p*	Total (*n* = 261) *M* (SD)	Boy (*n* = 139) *M* (SD)	Girl (*n* = 122) *M* (SD)	*p*			
Locomotor skills	46	36.096 (5.224)	35.982 (5.636)	36.234 (4.733)	0.808	37.838 (4.624)	36.557 (5.040)	39.266 (3.640)	<0.001	37.100 (5.292)	36.776 (4.931)	37.467 (5.673)	0.294	3.784	0.023	A<B
Object control skills	54	34.375 (6.579)	36.088 (6.495)	32.298 (6.125)	0.003	35.850 (6.612)	37.602 (6.356)	33.899 (6.376)	<0.001	39.720 (5.913)	40.863 (5.369)	38.418 (6.248)	0.001	35.035	<0.001	A<C B<C
Actual motor competence	100	70.471 (9.165)	72.070 (8.944)	68.532 (9.148)	0.049	73.688 (8.708)	74.159 (9.607)	73.165 (7.610)	0.462	76.820 (9.232)	77.640 (8.287)	75.885 (10.158)	0.126	19.487	<0.001	A<B<C
Perceived locomotor skills	4	3.542 (0.548)	3.608 (0.554)	3.461 (0.535)	0.174	3.450 (0.490)	3.396 (0.516)	3.510 (0.456)	0.132	3.291 (0.535)	3.265 (0.542)	3.321 (0.526)	0.399	10.101	<0.001	A>C B>C
Perceived object control skills	4	3.427 (0.603)	3.511 (0.595)	3.325 (0.604)	0.118	3.353 (0.499)	3.386 (0.485)	3.316 (0.515)	0.368	3.322 (0.532)	3.352 (0.545)	3.287 (0.482)	0.311	1.461	0.233	
Perceived motor competence	4	3.480 (0.539)	3.556 (0.534)	3.388 (0.536)	0.114	3.398 (0.451)	3.391 (0.448)	3.406 (0.457)	0.827	3.308 (0.490)	3.312 (0.506)	3.302 (0.474)	0.877	5.037	0.007	A>C
Physical activity	5	3.097 (1.014)	3.151 (1.050)	3.031 (0.975)	0.551	3.246 (0.857)	3.209 (0.901)	3.286 (0.810)	0.563	3.388 (0.759)	3.447 (0.783)	3.321 (0.727)	0.183	4.690	0.010	A<C

**Figure 1:**
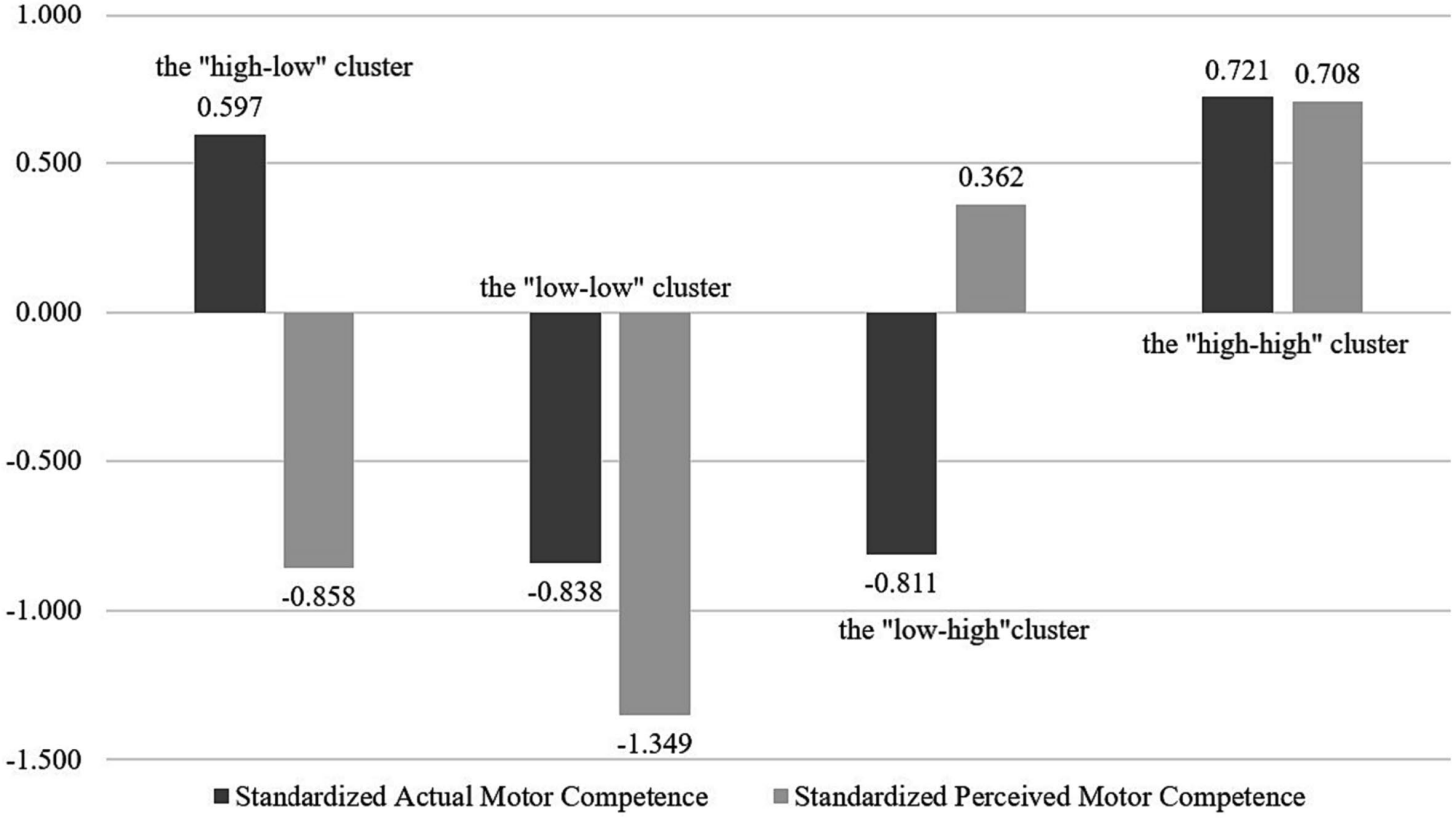
Four cluster solution based on standard scores for AMC and PMC.

Classifying the sample categories based on relative AMC (high vs. low) and PMC (high vs. low), it was found that four clusters of children with different MC profiles could be identified (e.g., Figure 1). Children in cluster 1 (*N* = 100, 19.46%) were found to have higher AMC and lower PMC compared to children in the other clusters, and thus named the “high-low” cluster. Children in cluster 2 (*N* = 63, 12.26%) had lower AMC and PMC compared to children in the other clusters, hence the name the “low-low” cluster. Children in cluster 3 (*N* = 151, 29.38%) were characterized by their lower AMC and higher PMC, hence the name the “low-high” cluster. Children in cluster 4 (*N* = 200, 38.91%) had higher AMC and PMC compared to the other clusters, hence the name the “high-high” cluster. Chi-square test indicated that boys and girls were equally represented in each cluster (ꭓ^2^ = 1.992, df = 1, *p* = 0.158). The four clusters were significantly different (*p* < 0.001) for both AMC and PMC (as shown in Table 3). In terms of AMC, significant differences were found between the “high-high” cluster (*M* = 81.355, SD = 8.575) and the “high-low” cluster (*M* = 80.190, SD = 4.735) on the one hand and the “low-high” cluster (*M* = 67.000, SD = 5.062) and the “low-low” cluster (*M* = 66.746, SD = 5.448) on the other hand. With respect to PMC, the “high-high” cluster had the highest mean score (*M* = 3.718, SD = 0.216), followed by the “low-high” cluster (*M* = 3.548, SD = 0.250), the “low-high” cluster (*M* = 2.948, SD = 0.268) and the “low-low” cluster (*M* = 2.706, SD = 0.283).

**Table 3:** Mean scores, standard errors and cluster comparisons for the four clusters (*N* = 514).

Variables	Cluster				*F*	*p*
	Cluster1: high-low	Cluster 2: low-low	Cluster 3: low-high	Cluster 4: high-high		
	*n* = 100, 19.46%	*n* = 63, 12.26%	*n* = 151, 29.38%	*n* = 200; 38.91%		
**Cluster dimensions (standard scores)**						
Actual motor competence	0.597 (0.505)	−0.838 (0.582)	−0.811 (0.540)	0.722 (0.540)	323.276	<0.001
Perceived motor competence	−0.858 (0.544)	−1.349 (0.576)	0.362 (0.509)	0.708 (0.440)	410.583	<0.001
**Cluster dimensions (raw scores)**						
Actual motor competence	80.190 (4.735)	66.746 (5.448)	67.000 (5.062)	81.355 (8.575)	323.276	<0.001
Perceived motor competence	2.948 (0.268)	2.706 (0.283)	3.548 (0.250)	3.718 (0.216)	410.583	<0.001

Values in parentheses are standard errors.

It could be seen from Table 4 that children with different MC profiles had significant differences in PA levels (*F* = 16.317, *p* < 0.001). From the average score of the PA level of each group, the “high-high” cluster (*M* = 3.588, SD = 0.825) had the highest PA level, and the “low-high” cluster (*M* = 3.212, SD = 0.905) had the second highest PA level. The “high-low” cluster (*M* = 3.113, SD = 0.658) had the third highest level of PA, and the “low-low” cluster (*M* = 2.876, SD = 0.781) had the lowest. The “high-high” cluster had significantly higher levels of PA than the other three groups, and the “low-high” cluster had significantly higher levels of PA than the “low-low” cluster.

**Table 4:** Mean scores, standard errors and cluster comparisons for the four clusters (*n* = 514): PA.

Variable	Cluster				*F*	*p*	Multiple comparisons (LSD)
	Cluster1: high-low (1)	Cluster 2: low-low (2)	Cluster 3: low-high (3)	Cluster 4: high-high (4)			
Physical activity	3.113 (0.658)	2.876 (0.781)	3.212 (0.905)	3.588 (0.825)	16.317	<0.001	(1)<(4) (2)<(3)<(4)

Values in parentheses are standard errors. All values are controlled for sex and age.

## 4 Discussion

This study investigated the AMC, PMC and PA level of Chinese school-age children, adopted a person-centered approach to explore the relationship between AMC and PMC of Chinese school-age children, identified children groups based on different MC profiles, and explored how different groups of children differed in PA levels.

This study found that the locomotor skills of girls aged 8–9 years were significantly higher than that of boys, the object control skills of girls at all ages were significantly lower than that of boys, and the AMC of boys aged 6–7 years was higher than that of girls, which was consistent with previous research results (52–55). Children’s FMS produce certain gender differences, which could be related to socio-cultural and environmental factors. During childhood, boys were more likely to choose ball games and equipment, while girls were more likely to choose dance and rhythmic gymnastics. In addition, some children, especially girls, showed relatively unfamiliar use of equipment during the test, which could lead to a certain impact on the test effect. Ning et al. (56) also found that boys’ object control skills were significantly better than girls’ among Chinese pre-school children aged 4–7, indicating that boys’ object control skills were already better than girls’ from early childhood.

This study did not find gender differences in PMC and PA levels among school-age children. In fact, no conclusion had been reached on gender differences in children’s PMC. For example, Diao et al. (16) found that there was no significant gender difference in children’s self-perception of FMS in pre-school age (4–6 years old), but school-age (7–9 years old) boys’ self-perception of FMS was significantly better than that of girls. LeGear et al. (57) found that girls’ perceived physical abilities were higher than boys’. The inconsistent results of gender differences in PMC could be attributed to the selection of samples from different regions. Intuitively, the results of the present study may promote additional study in order to provide extended knowledge on gender differences in PMC. As for PA, most studies had found that boys engaged in more moderate-to-vigorous PA than girls (58–60). The reason why there was no gender difference in children’s PA level in this study could be that this study collected data by means of self-report. Children generally had a high perception of their own athletic competence, and 3–5 physical education classes were generally offered in schools every week, and children had more opportunities for PA in school. Therefore, both boys and girls could report more time and frequency of PA.

This study found that the AMC and PA level of school-age children had obvious age characteristics, which was consistent with previous studies and in line with the growth law of children to a certain extent (61–63). According to Newell’s constraint model, the outcome of an individual’s MC development is the result of a combination of personal, task, and environmental factors (64). Influenced by family, community, and school environments, Children’s motor experiences were constantly being enhanced and developed, progressively developing higher levels of AMC, and therefore being competent to engage in more PA. In addition, this study found that PMC of 6–7 years old children was significantly higher than that of 10–12 years old children. Previous literature showed that school-age children were in a period of rapid physical and mental development, and PMC would change rapidly with the increase of age (39). In early childhood, children’s perception accuracy of their own competence was poor, and they often overestimated their own competence. However, with the growth of children’s age and the continuous enrichment of personal experience, children’s self-perception of competence would gradually converge with the real competence.

Based on the characteristics of children’s MC, cluster analysis identified four groups of children. Two of the groups had corresponding levels of AMC and PMC, 12.26% of the children had relatively low levels of both AMC and PMC (the “low-low” cluster), and 38.91% of the children had relatively high AMC and PMC levels (the “high-high” cluster). Additionally, this study identified two groups of children with inconsistent levels of AMC and PMC, with some children (19.46%) having high levels of AMC but exhibiting low levels of PMC (the “high-low” cluster), and a larger portion of children (29.38%) showed relatively low AMC and high PMC (the “low-high” cluster). This was consistent with the results of a study of Belgian children aged 7–11 years, which also identified four MC-based profiles (22). Almost half of the children showed inconsistent levels of AMC and PMC, and one-third of the children overestimated their MC, suggesting that although children had more accurate cognitive and evaluative abilities in the middle and late stages than in the early stages (65), they still tended to overestimate their AMC.

The results of the study further indicated that there were differences in the PA levels of children with different MC profiles. Specifically, children in the “high-high” cluster had the highest level of PA, while children in the “low-low” cluster exhibited the lowest level of PA, which confirmed the hypothesis of the present study and was consistent with the results of existing studies (20, 35). The significant difference in PA levels between children with high AMC (“high-high” cluster) and children with low AMC (i.e., “low-low” cluster, “low-high” cluster) suggested that AMC was an important factor that influences children’s participation in PA, highlighting the importance of promoting children’s participation in sports and PA through the development of children’s AMC (66). Bolger et al. (67) concluded that children with higher AMC were more likely to participate in organized sports activities, children could gain more knowledge about FMS from physical education teachers or coaches and promote their PA intensity. In addition, this study found that children with higher levels of PMC had higher levels of PA when their AMC levels remained consistent. This suggested that PMC also played an important role in facilitating children’s PA, and that children needed to have a sense of belief in their competence to accomplish motor skills in order to be motivated to engage in PA. Competence Motivation Theory suggested that when individuals attempted to learn a motor skill, they derived “enjoyment” from learning it if they felt competent to do so, and that the recognition and approval of peers, teachers, and parents during the learning process accelerated the development of good PMC (2, 68). Once they had learned these motor skills, this PMC would motivate them to continue to participate in sport. For this reason, special attention needed to be paid to context-specific movement activities that were developmentally appropriate and that promoted the development of children’s AMC and PMC, which could help to promote children’s active participation in PA (69). While we focused on improving children’s AMC through intervention programs and measures, we should also pay attention to the development of children’s PMC. On the one hand, novel ways of PA, such as somatic games, could be introduced to arouse children’s interest and mobilize their motivation; on the other hand, while shaping a relaxing and enjoyable physical education learning environment, reinforcement should be given to children to emphasize their successes, efforts and progress.

Nevertheless, it seemed important to highlight the limitations. First, although the sample size of this study was large and distributed across grades and urban and rural areas, only school-aged children in Beijing were selected as the target population, and the sampling should be expanded in the future to study the characteristics of children’s MC and its relationship with PA in different areas, and future studies were required to further compare the performance of AMC and PMC and the relationship between AMC and PMC in children in rural and urban areas. Second, this study conducted a cross-sectional study, which provided a new perspective by adopting a person-centered approach, but it was unable to determine the causal relationship between children’s AMC, PMC, and PA; therefore, future tracking studies should be conducted to determine the dynamic developmental trajectory among the three variables as children’s age changes. Finally, the present study tested children’s AMC using a well-established measurement tool and measured children’s PMC using a scale corresponding to the TGMD-3 items, which reduced errors due to inconsistencies in the instrument items; however, this study used a self-report questionnaire, which had a certain degree of subjectivity, which leaded to the possibility that the results could have been overestimated or underestimated. Therefore, the use of more objective measurement tools and methods to collect data would be a future endeavor to continue the study, making the results more reliable.

## 5 Conclusion

The present study revealed the AMC and PMC characteristics of school children from Beijing City, as well as explored the differences in PA levels of children based on two different MC characteristics. The results of this study showed that there were some gender differences in children’s AMC, with girls having better locomotor skills than boys, while boys performed better than girls in object control skills and overall AMC, but no gender differences were found in PMC and PA. In addition, AMC, PMC, and PA all had certain age characteristics that were consistent with the growth and development of children, AMC and PA improved to some extent with age, while children’s PMC declined and their perception of MC became accurate. The present study identified four groups of children based on different MC profiles, and nearly half of the children showed inconsistent AMC and PMC, with children with high AMC having low PMC and children with low AMC having high PMC. Children with different MC characteristics had different levels of PA, to be specific, children with high AMC and high PMC showed higher levels of PA, which provided an intervention perspective for promoting active PA in children. Families, schools, and communities should collaborate to help children acquire good FMS at an early age, improve their levels of AMC, and develop good PMC and active lifestyles.

## Data availability statement

The raw data supporting the conclusions of this article will be made available by the authors, without undue reservation.

## Ethics statement

Written informed consent was obtained from the individual(s), and minor(s)’ legal guardian/next of kin, for the publication of any potentially identifiable images or data included in this article.

## Author contributions

HC: Conceptualization, Data curation, Formal analysis, Funding acquisition, Investigation, Methodology, Project administration, Resources, Software, Supervision, Validation, Visualization, Writing – original draft. RX: Conceptualization, Data curation, Formal analysis, Funding acquisition, Investigation, Methodology, Project administration, Resources, Software, Supervision, Validation, Visualization, Writing – original draft. LY: Investigation, Resources, Supervision, Visualization, Writing – review & editing. MM: Data collection, Formatting, Writing – review & editing. BH: Revision, Funding, Methodology, Writing – review & editing.
